# Supplementary material for: Identification of Genes Associated with Gut Microbiota Metabolites in Type 2 Diabetes Mellitus Complicated by Acute Cerebral Infarction Based on Bioinformatics and Mendelian Randomization
Source: Biomedicines. 2026 Oct 17;14(10):2371. doi: 10.3390/biomedicines14102371 (PMC13702449; doi:10.3390/biomedicines14102371)
Supplement: Supplementary file 1 [file biomedicines-14-02371-s001.zip › Figure S3/genus.CandidatusSoleaferrea.id.11350 ACI.pdf]

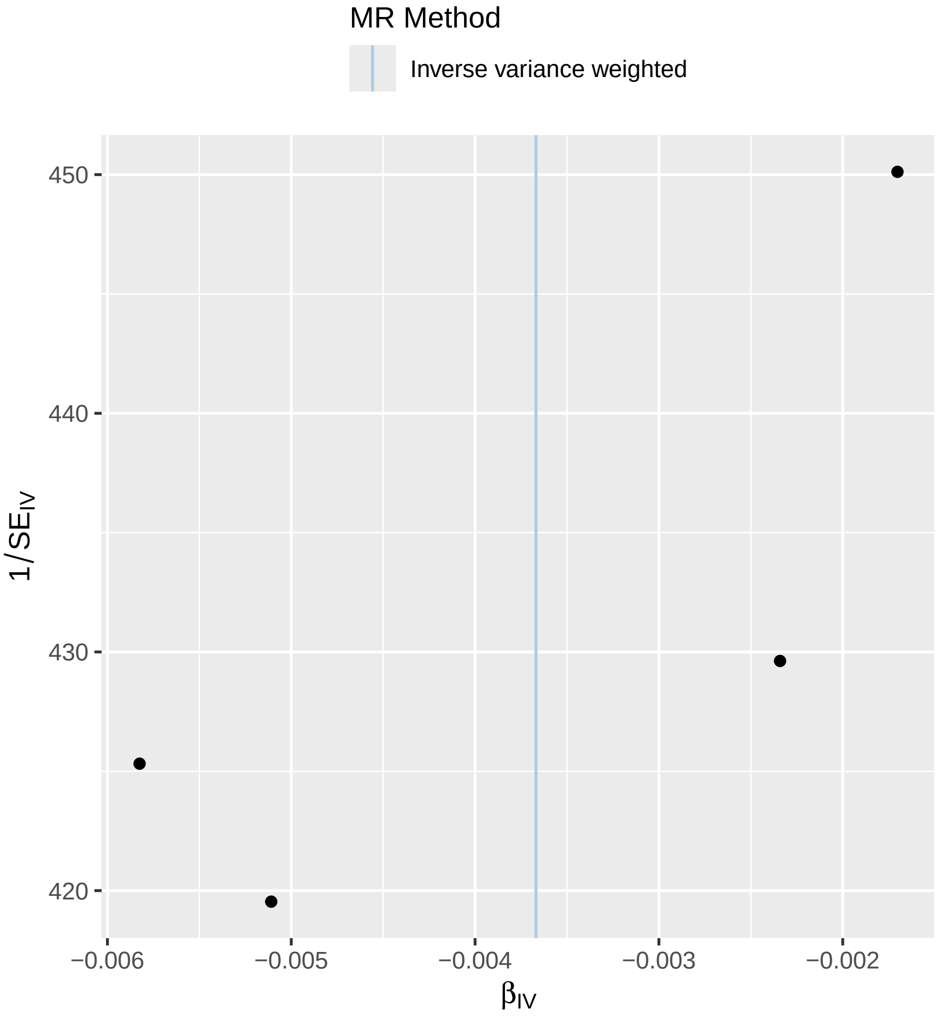
MR Method
Inverse variance weighted
450
440
430
420
$1/SE_{IV}$
−0.006
−0.005
−0.004
−0.003
−0.002
β_{IV}
